# Three-Dimensional Microfluidic Capillary Device for Rapid and Multiplexed Immunoassays in Whole Blood

**DOI:** 10.1021/acssensors.4c00153

**Published:** 2024-04-30

**Authors:** Thomas Mortelmans, Balz Marty, Dimitrios Kazazis, Celestino Padeste, Xiaodan Li, Yasin Ekinci

**Affiliations:** † Laboratory for X-ray Nanoscience and Technologies, 5232 Villigen, Switzerland; ‡ Laboratory of Nanoscale Biology, Paul Scherrer Institute, 5232 Villigen, Switzerland; § Laboratory of Biomolecular Research, Paul Scherrer Institute, 5232 Villigen, Switzerland; ∥ Swiss Nanoscience Institute, University of Basel, 4056 Basel, Switzerland

**Keywords:** immunosensors, multiplexing, microfluidics, blood, diagnostics

## Abstract

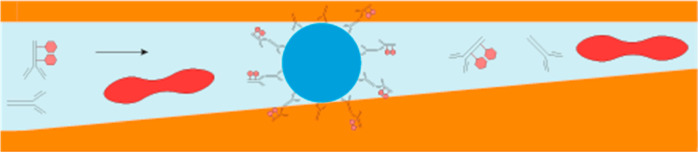

In this study, we demonstrate whole blood immunoassays using a microfluidic device optimized for conducting rapid and multiplexed fluorescence-linked immunoassays. The device is capable of handling whole blood samples without any preparatory treatment. The three-dimensional channels in poly(methyl methacrylate) are designed to passively load bodily fluids and, due to their linearly tapered profile, facilitate size-dependent immobilization of biofunctionalized particles. The channel geometry is optimized to allow for the unimpeded flow of cellular constituents such as red blood cells (RBCs). Additionally, to make the device easier to operate, the biofunctionalized particles are pretrapped in a first step, and the channel is dried under vacuum, after which it can be loaded with the biological sample. This novel approach and design eliminated the need for traditionally laborious steps such as filtering, incubation, and washing steps, thereby substantially simplifying the immunoassay procedures. Moreover, by leveraging the shallow device dimensions, we show that sample loading to read-out is possible within 5 min. Our results also show that the presence of RBCs does not compromise the sensitivity of the assays when compared to those performed in a pure buffer solution. This highlights the practical adaptability of the device for simple and rapid whole-blood assays. Lastly, we demonstrate the device’s multiplexing capability by pretrapping particles of different sizes, each functionalized with a different antigen, thus enabling the performance of multiplexed on-chip whole-blood immunoassays, showcasing the device’s versatility and effectiveness toward low-cost, simple, and multiplexed sensing of biomarkers and pathogens directly in whole blood.

Immunoassays, employing antibody-based detection methods for analytes of low concentrations,^1^ are indispensable tools in diverse fields such as pharmaceutical applications, biological research,^2^ and diagnostics.^3^ Despite their wide-ranging utility, these assays often present challenges, including significant time and lab infrastructure requirements and the necessity for substantial sample volumes, which may hinder their effectiveness, in particular in resource-limited settings or situations requiring prompt diagnosis, such as acute cases of sepsis.^4^ For instance, laboratory-based enzyme-linked immunosorbent (ELISA) assays allow for a high degree of sensitivity and biomarker quantification but require a lengthy time until read-out. A classical sandwich ELISA requires overnight plate functionalization and numerous consecutive sample loading steps with extensive intermediate washing, taking 5–7 h for an experienced operator to perform.^5^ Moreover, ELISAs require a minimum sample volume of 100 μL per well or condition when using conventional 96-well plates,^6^ whereas in many fields, such as structural biology^7^ or high-throughput drug discovery,^8^ sample volumes are a limiting factor. Alternate methods that can provide rapid and cost-effective diagnosis are therefore needed, particularly in point-of-care (POC) settings. For example, lateral flow assays (LFAs)^9−11^ have underscored the need for rapid and low-cost diagnostic methods through their role in combating the global outbreak of COVID-19.^12,13^ However, they typically provide only binary outcomes and are limited in sensitivity.^47^ Therefore, there is a pressing need in both POC and laboratory settings for rapid, sensitive, and low-cost diagnostic devices that are simple to use and compatible with minimal sample volumes.

In general, immunoassays can be employed to investigate the presence of analytes in a wide variety of biofluids, of which plasma is among the most commonly used. Comprising 55% of whole blood, plasma contains a large variety of proteins that are essential to maintain bodily hemostasis, and it serves as an invaluable source of biomarkers to assess the patient’s status or diagnostics, such as revealing infections,^14^ autoimmune diseases,^15^ and many other disorders.^16−18^ The remaining volume of blood consists of cells, of which the most abundant are the red blood cells (RBCs). These are biconcave disks with thicknesses of 2.5 μm at the edges and 1 μm in the center. They are rich in hemoglobin, which gives them their distinctive red color.^19^ As the read-out mechanism of immunoassays is most often colorimetric or fluorescent-based, the intense red color of RBCs introduces severe optical or colorimetric interference effects, resulting in significant masking and disturbance of the signal from the target analyte.^20^ Therefore, numerous methods have been developed to remove the cellular material, such as centrifugation,^21^ agglutination,^22^ and whole blood filtration.^9,23^ In the case of LFAs, the last two are often combined in membrane-based dead-end filtration techniques.^24^ A major downside of using such membranes is that they do not only retain RBCs but also partially retain the potential plasma biomarkers due to the electrostatic and hydrophobic effects, effectively reducing the concentration before they reach the testing area.^25,26^ Furthermore, such filtration could possibly cause hemolysis, leading to reduced precision of analyte concentration measurement.^27^ In addition, the flow properties of LFAs are largely governed by the employed membrane, which is subject to significant supplier variability.^28^ They also restrict easy flow customization, hampering the multiplexing potential of such devices.

Micro- and nanofluidic systems offer a promising alternative due to their tunable flow properties and incorporation of various functionalities and fluidic elements on a single chip, such as resistors, valves, multiple inlets, and mixers,^29^ Nevertheless, the integration of filter membranes into micro- and nanofluidic systems is not straightforward and poses significant fabrication challenges. To overcome this, it is possible to integrate on-chip blood plasma separation by exploiting various fluidic principles, such as deterministic lateral displacement,^30^ the Zweifach–Fung effect,^31^ or inertia-based methods.^32,33^ Yet, this necessitates meticulous control over the flow rate by means of active pumping, significantly hampering the applicability of such assays in a POC setting.^34^ Additionally, as with LFAs, conventional microfluidic POC devices still require cumbersome prepatterning of the functional area,^35,36^ which limits their up scalability.

Here, we report a poly(methyl methacrylate) (PMMA), i.e., plexiglass, three-dimensional (3D) microfluidic device, which enables performing fluorescence-linked immunosorbent on-particle and on-chip immunoassays directly in whole blood, without the need for sample preprocessing, active pumping strategies, or external device prepatterning. The device has a 3D channel profile which enables size-dependent immobilization of biofunctionalized particles.^3,37^ In the 3D channel, the functionalized particles are preimmobilized at a given position in the channel, and whole blood is subsequently flushed through the channel in a second loading step. We outline the optimization of the channel topography to efficiently pretrap the particles and ensure the easy flow of whole blood through the device. We, then, use a channel profile that enables whole blood flow to perform on-chip immunoassays. We also demonstrate multiplexing capabilities by pretrapping two sizes of biofunctionalized particles against different antigen targets. Due to size-dependent particle separation, the deposition of such particle-testing lines is straightforward and completely passively performed, without necessitating external infrastructure, in comparison to LFA fabrication.^38,39^ The device is purely driven by capillary forces, and therefore, it is self-powered and simple to use, paving the way for its extensive applications in POC settings.

## Methods

### Device Design and Grayscale e-Beam Patterning

**Figure 1:**
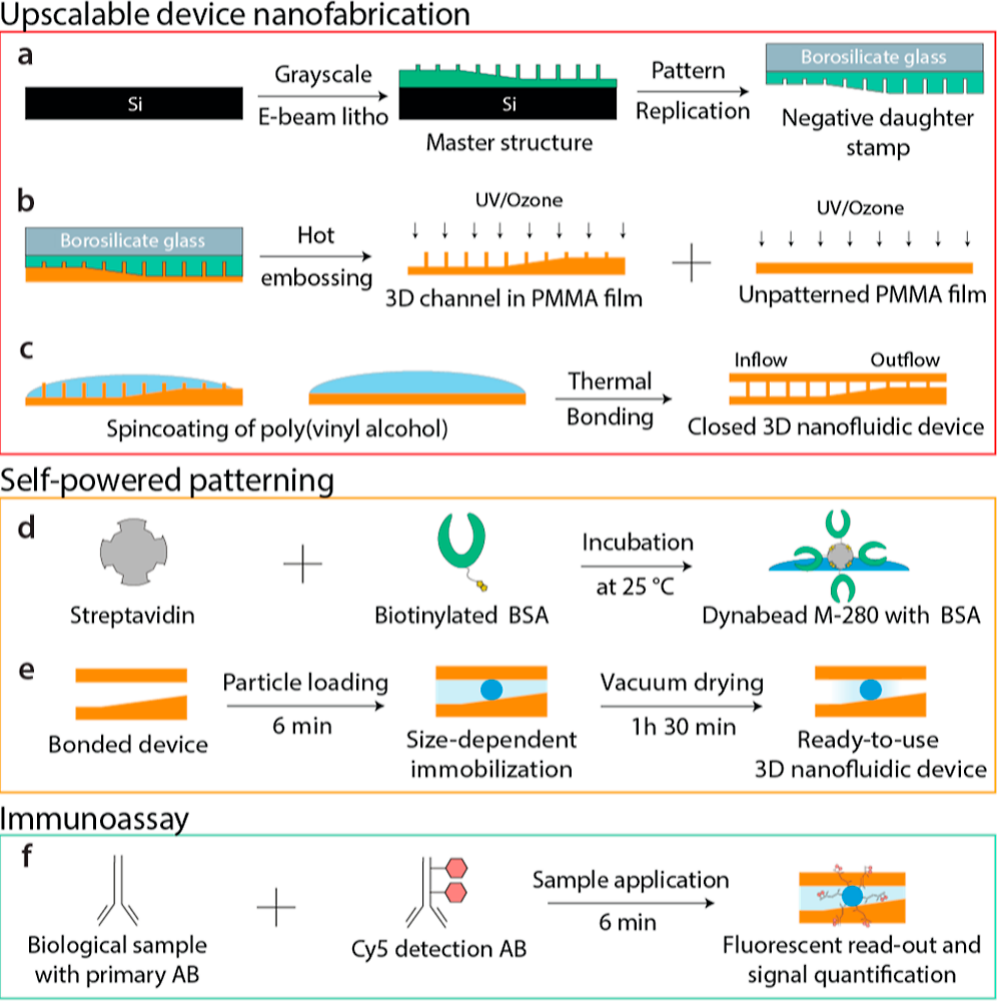
Overview of the 3D nanofluidic immunoassay process. (a) Manufacturing of the master structure through grayscale e-beam lithography in PMMA 950 K, spin-coated on a silicon wafer. The master structure was subject to pattern replication to fabricate a negative daughter stamp for hot embossing. (b) Hot-embossing of a flat PMMA sheet with the negative daughter stamp, followed by UV/ozone-activation at 172 nm. (c) Functionalization of the PMMA surface with PVA through spin-coating and spin-washing. The two PMMA films were aligned and thermally bonded. (d) Functionalization of 2.8 μm particles with BSA via the high-affinity interaction of streptavidin and biotin. Similar functionalization was performed for horseradish peroxidase (HRP) particles. (e) Size-dependent immobilization of biofunctionalized particles and vacuum drying to finalize self-powered patterning of testing lines. (f) One drop immunoassay by mixing the sample under investigation with detection antibodies and loading it into the device through capillary forces.

The devices consist of two PMMA, i.e., plexiglass, substrates that are bonded together to realize the 3D channels as well as the inlets and outlets. To elaborate, a 1 mm-thick plexiglass sheet is patterned via hot embossing, utilizing master stamps that are fabricated using e-beam lithography (Figure 1a,b). The structured PMMA is then bonded to a 200 μm-thick optical-grade PMMA sheet to form micro- and nanochannels (Figure 1c).

The master stamp was designed using the open-source GDS II-based PHIDL Python module.^40^ As GDS II files are inherently two-dimensional, the 3D topography of the device’s structure was defined by using a separate layer per distinct height level. In total, 1000 layers or gray levels were used. A 4" Si wafer is patterned to contain 2 × 9 arrays of fluidic channels. Each array of 9 channels was considered to be one fluidic chip. A more in-depth description of the grayscale e-beam lithography fabrication procedure is outlined elsewhere.^37,41^

### Fabrication of Daughter Stamps and Devices with Hot Embossing

After patterning with e-beam lithography, a negative copy of the master stamp is used for hot embossing of the microfluidic structures. For the production of a negative copy, a 4" borosilicate wafer was cleaned in acetone for 30 s and subsequently in isopropyl alcohol for 30 s. The wafer was then blow-dried with nitrogen, followed by the activation of the surface with oxygen plasma using PlasmaLab 80 RIE (Oxford Instruments) at 20 W with a pressure of 20 mTorr for 1 min. The activated wafer was spin-coated with an adhesion promoter (OrmoPrime, Microresist) at 4500 rpm for 45 s. The spin-coated wafer was baked at 150 °C for 5 min. The next stage involves pipetting 1 mL of a photocurable polymer (GMN PS-90, Optool) onto the wafer, which was then carefully placed on top of the master stamp. The photocurable polymer was allowed to spread between the glass wafer and the master stamp for at least 20 min. Subsequently, the polymer was exposed to UV light at a wavelength of 365 nm and with a power of 300 mW/cm^2^ for 6 min. After this curing, the photocurable polymer, due to its inherent antiadhesive properties, allows for the hardened polymer (referred to as the daughter stamp) to be easily removed from the master stamp.

The daughter stamp was used to hot emboss the negative structures into a 1 mm thick PMMA sheet. This process involves placing the daughter stamp and the PMMA sheet into a hot embossing chamber (Jenoptik Hex 03). A flat 4″ silicon wafer, treated with an antiadhesion coating, was placed on the backside of the PMMA sheet. This is followed by a poly(amide)–poly(dimethylsiloxane)–poly(amide) sandwich to equalize the pressure on the PMMA’s surface. Initially, a touch force of 300 N was applied, and the imprinting chamber was heated to 160 °C at a rate of 9 °C/min. The force was subsequently increased to 10 000 N and maintained at this level for 15 min. Finally, the chamber was cooled down to a demolding temperature of 60 °C before the PMMA sheet was removed from the chamber (Figure 1b).

The topography of the fabricated devices was investigated via optical and contact profilometry using a Keyence VK-X1100 at a 150× magnification and a Veeco Dektak 150, equipped with a 2.5 μm stylus, respectively. On a 4″ PMMA, two arrays of 9 devices are obtained, which were diced into the single devices. The fabrication process described herein allows for cost-effective device production. The most expensive step, the fabrication of the master stamp with e-beam lithography, yields a large number of daughter stamps, which, in turn, allow for the production of many devices. This two-step pattern transfer method significantly reduces the overall fabrication cost.

### Device Functionalization and Bonding

Following the hot-embossing step, the surface of the PMMA sheets was activated with oxygen plasma at a power of 80 W and a pressure of 0.8 mbar for 20 s (Tepla AG). This process temporarily renders the PMMA surface hydrophilic, enabling the spin-coating of a water-soluble protection layer, which is 10% dextran in Milli-Q (66 kDa Roth Industries) that is spin-coated at 3000 rpm for 60 s. The PMMA sheet was then cut into two chips, each containing 9 devices. Afterward, the protective layer was dissolved by immersing the single chips in deionized water for 15 min. To ensure that the PMMA was fully dried before further processing, the chips were blow-dried with nitrogen and then placed in vacuum for at least 10 min.

For device assembly, the patterned PMMA was activated together with an unpatterned, 200 μm-thick, optical-grade PMMA film by UV/ozone irradiation at a wavelength of 172 nm for 30 s. This process reduces the molecular weight of the polymer on the surface and thereby lowers the glass transition temperature relative to the bulk material.^8,42^ The surfaces of both PMMA films were spin-coated with poly(vinyl alcohol) [PVA; 0.5% in phosphate-buffered saline (PBS) with a pH of 7.4] at 2000 rpm for 1 min to prevent unspecific interactions between biomolecules and the surface of the PMMA.^43^ Any excess PVA was removed by spin-washing with deionized water at 2000 rpm for 1 min. Once coated, both PMMA surfaces were thermally bonded at 750 N and 45 °C for 1 min in a Jenoptix Hex 03 press (Figure 1c). This comprehensive process ensures the precise and reliable assembly of the devices.

### Capillary Filling as a Function of the Wedge Profile

The fluidic filling of the capillary pump (CP) was investigated by using a Leica DMi8 microscope equipped with a Leica-K5-14400713 detector. A 5× objective with a numerical aperture of 0.12 was used to have a large field-of-view and an emission filter at 527 nm. The influence of the wedge profile and the blood concentration on the flow properties of the 3D microfluidic device were investigated by using nine unique channel profiles, in combination with three concentrations of ethylenediaminetetraacetic acid (EDTA)-treated rabbit whole blood (undiluted, 1:2, 1:40; Envion). To simplify the tracking of the fluid, a fluorescent dye (ATTO488) was added to all three solutions with a concentration of 125 μM. During the loading phase of the device, time-lapse series were acquired to monitor the evolution of flow velocity during filling. Also, 5 min after loading the device, a stitched image was taken to assess the filling factor of the CP. The images were analyzed with a custom Python script in which image segmentation was performed to identify the number of fluorescent pixels and to determine the corresponding volume. These data sets were subsequently used to obtain the average flow rate during capillary filling.

### Bovine Serum Albumin Particle Functionalization and Pretrapping

Streptavidin-coated 2.8 μm magnetic particles (Dynaparticles M-280 Streptavidin, Thermo Fisher Scientific, 11205D) were washed three times in PBS. Subsequently, the targeted concentration of particles was resuspended in a PBS solution containing 160 pmol of biotinylated bovine serum albumin (BSA) per μg of particles (Pierce BSA, Biotinylated, Thermo Fisher Scientific, 29130). The protein–particle mixture was incubated at 600 rpm and 25 °C for 2 h with periodic vortexing every 30 min and finally washed three times with PBS (Figure 1d). The functionalized particles were diluted in deionized water and applied to the inlet of the microfluidic device so that the trapped particles formed a discernible line in the trapping region upon complete filling of the CP. The filled devices were dried in vacuum for 1.5 h to ensure the complete removal of the fluid (Figure 1e).

### Time Lapse of the BSA Immunoassay

To investigate the time evolution of the signal when loading the device, a 2 μL droplet of PBS, containing 130 nM diluted anti-BSA antibody (Bethyl Laboratories; bovine albumin polyclonal antibody, A10-127A) and 50 nM Cy5 donkey antirabbit antibody, was applied onto the inlet of the device. The fluorescence signal of the particles was then monitored over a period of 900 s, with 45 s dark intervals between the readings to minimize photobleaching. The image acquisition was performed by using a bright time duration of 2 s. For a negative control, a PBS solution containing only Cy5 donkey antirabbit antibody was used and monitored over the same time frame and with the same bright period as the positive sample.

### Limit-of-Detection of BSA Antibodies

Devices with a minimum channel height of 2.2 μm and containing pretrapped 2.8 μm BSA-functionalized particles were employed to perform a proof-of-principle immunoassay in diluted whole blood (1:40). More specifically, the diluted, EDTA-treated rabbit whole blood (Envion) was spiked with varying concentrations of rabbit anti-BSA antibodies (Bethyl Laboratories; bovine albumin polyclonal antibody, A10-127A). As secondary detection antibodies, Cy5-conjugated donkey antirabbit antibodies (JacksonImmuno, AB_2340607) were added to the spiked samples at 50 nM immediately before filling the capillary devices. The experiment was repeated 3 times for each anti-BSA antibody concentration. Six minutes after device filling, a fluorescence and a bright-field image of the trapping region were captured. The latter enabled easy identification of the particles via the same Python script described in the previous section. As a comparison, the same experiment was performed in PBS without whole blood (Figure 1f). To determine the fluorescence intensity, the median fluorescence per particle over an ensemble of particles is used, which was obtained by fitting a sigmoidal function to the fluorescence intensity profile of individual particles. The resulting fit was used to calculate the limit-of-detection (LOD) as the median signal of the control sample without anti-BSA antibodies plus 3 times its standard deviation.

### Multiplexed Detection of HRP and BSA in Whole Blood

The 3D profile of the device was fine-tuned to a minimum channel height of 1.9 μm to enable the multiplexed detection of two different antigens in a proof-of-principle setting. For this, alongside the 2.8 μm BSA-functionalized particles, 2 μm particles were included, which (Creative Diagnostics, WHM-G187) were functionalized using the bead functionalization protocol described earlier by incubating them in a PBS solution containing 25 pmol of biotinylated HRP (Thermo Fisher Scientific, 29139) per μg of particles. After functionalization, the particles were incubated with 0.9 mg/mL free biotin (Thermo Fisher Scientific, 29129) at room temperature for 30 min at 600 rpm to saturate all available streptavidin–biotin binding pockets to reduce cross-particle aggregation. Then, both particle suspensions were added together and pretrapped in the device as previously described. Subsequently, 50 nM Cy5 donkey antirabbit antibody was added to EDTA-treated rabbit whole blood (1:40 in PBS). The latter was spiked with rabbit anti-BSA or rabbit anti-HRP at 130 nM to showcase different immunological outcomes. Immediately afterward, a 2 μL droplet of the solution was applied to the inlet of the capillary microfluidic device, and the device was left for an incubation of 6 min. The particle fluorescence at the relevant trapping lines was imaged using a Leica DMi8 equipped with a 40× objective (NA: 0.95) in combination with an emission filter cube for a wavelength of 700 nm. The fluorescence quantification was done by using a custom Python image analysis script. The script used the scikit-image^44^ module to identify the relevant fluorescent pixels from the acquired bright-field image in which fluorescent beads are identified. The collected fluorescence images underwent a rolling-ball background correction, as did the previously obtained particle positions. Afterward, we performed rolling-ball background subtraction and equalization with a top-hat filter. Lastly, NumPy^45^ and Pandas^46^ were used to calculate the mean signal in the region of interest.

## Results and Discussion

### Design of a Capillary Device with a 3D Profile

In conventional microfluidic devices fabricated through binary lithography, the channel heights remain constant. Grayscale lithography, on the other hand, allows precise modulation of the channel topography, offering an additional degree of freedom in design, which considerably increases the possibility of fluid manipulations. With the ability to vary the channel height inside a microfluidic device, many new functionalities, such as advanced flow focusing^32^ and on-chip particle size determination,^47^ can be obtained.

**Figure 2:**
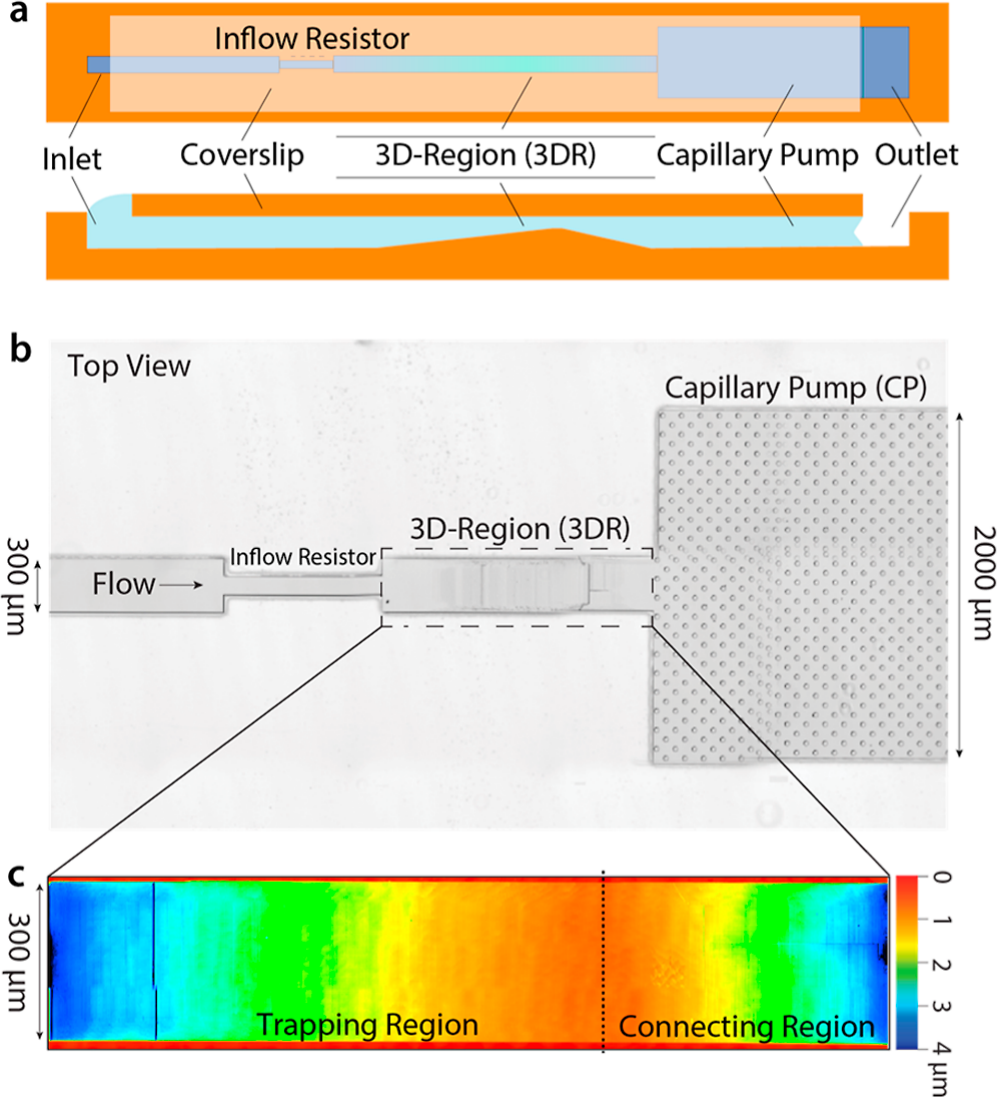
3D microfluidic device geometry. (a) Schematic showing the different microfluidic components of the capillary device. The top section shows a top view, and the bottom section is a cross-section to highlight the change in channel topography. (b) Bright-field image of a bonded microfluidic device, showing the flow direction as well as the three-dimensional region (3DR). (c) Confocal micrograph of the three-dimensional topography in the 3DR of a device with an outflow height of 800 nm.

We have previously leveraged the topographical variations inside fluidic channels to design a capillary-driven device capable of sterically trapping particles in the submicron regime. This effect was exploited to enable multiplexed antibody detection in patient serum.^37^ However, the previously reported process required ready-to-use serum and several external sample processing steps, including serum incubation and secondary antibody labeling. Moreover, due to the submicrometer channel height, the device was incompatible with whole blood samples. To overcome this, the device geometry was adapted, resulting in a self-powered 3D microfluidic device comprising an inlet, an inflow resistor (I.R.), a fine-tuned 3D region (3DR), and a CP (Figure 2a).

In this device, biofunctionalized particles are first size-dependently immobilized, and then the device is vacuum-dried. This enables on-chip immunoassays without any incubation steps outside of the device. Once dried, a 2 μL sample droplet is applied at a 300 μm inflow channel, which has a channel height of 4 μm (Figure 2b—left). Here, due to surface tension, the sample will be aspirated into the channel. Subsequently, the sample will flow through an I.R., which is 100 μm wide and 500 μm long. These dimensions increase the fluidic resistance, ensure a more homogeneous filling front, and thereby avoid the formation of bubbles in the channels.^29^ The I.R. is followed by the 3DR, which features two sections with distinct topographies. In the first section, the channel height gradually decreases over 1 mm to ensure size-dependent immobilization of biofunctionalized particles. The second section of the 3DR transitions the channel height back to 4 μm over a distance of 0.5 mm (Figure 2c) and links the active region of the device to a CP (Figure 2b—right). The CP is 4 μm deep, 2 mm wide, and 22.5 mm long, providing a total filling capacity of ∼150 nL. It contains pillars with a diameter of 30 μm and a pitch of 75 μm that contribute to the realization of a controlled flow.^5,29^

### Optimization of the 3D Channel Profile for Unhindered Flow of Whole Blood

**Figure 3:**
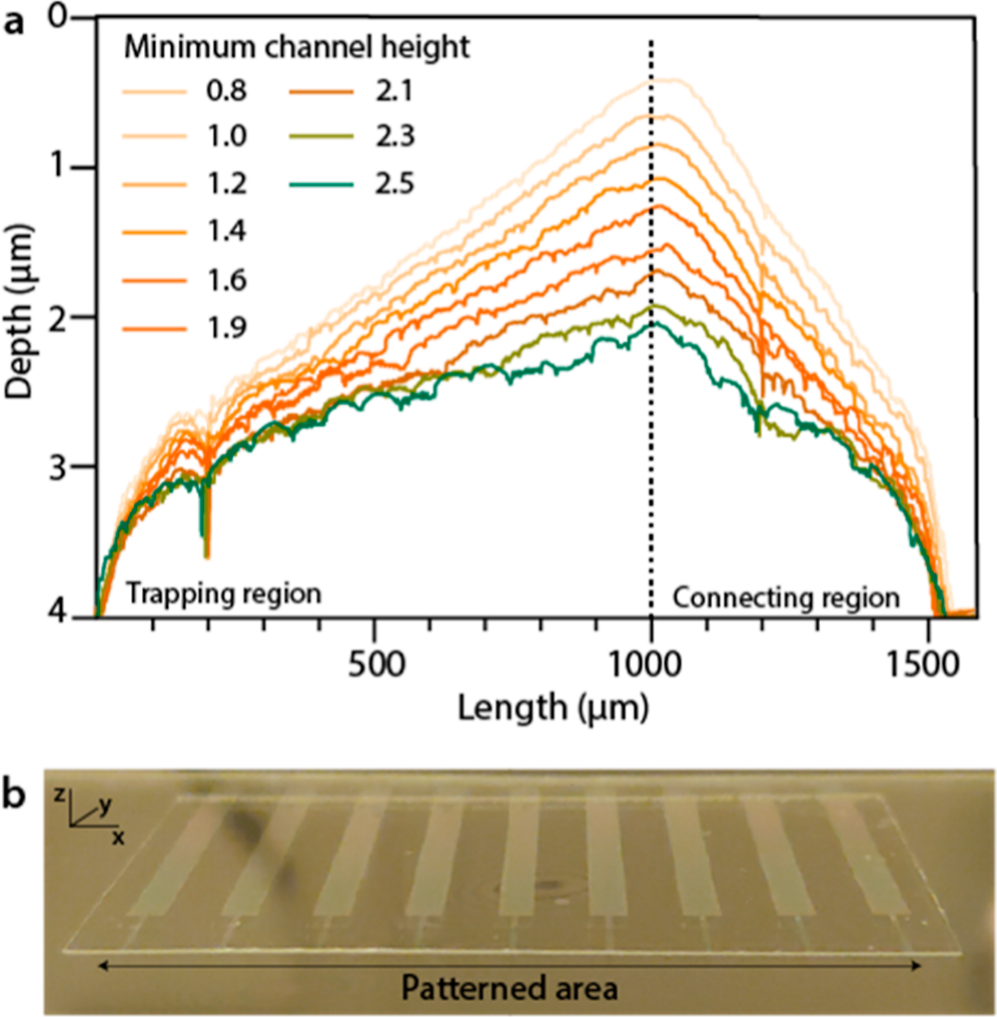
On-chip height screening. (a) Profiles of 9 different wedge topographies which were used to find the ideal outflow height. The trapping region indicates where the particle immobilization takes place. The connecting region is representative of the tapering that connects the trapping region with the CP. (b) Photograph showing a PMMA-based chip containing 9 different devices with different channel topographies in the patterned area. Each CP has a width of 2 mm.

To achieve on-chip whole blood assays in a straightforward and simple manner, it is of paramount importance that RBCs do not get halted in the 3DR, wherein optical readout is performed, and the presence of RBCs could disturb the measurement or would cause clogging and hinder the flow. Therefore, identifying the optimal height profile of the device that prevents RBC blockage is critical. To this end, we patterned nine devices with varied 3DRs on the same chip. Specifically, we systematically reduced the shallowest point of the channels by about 0.2 μm increments while maintaining a maximum depth of 4 μm in the inflow section (Figure 3a). These nine devices were patterned on the same chip by grayscale e-beam lithography, followed by hot embossing into a PMMA sheet (Figure 3b).^37^

**Figure 4:**
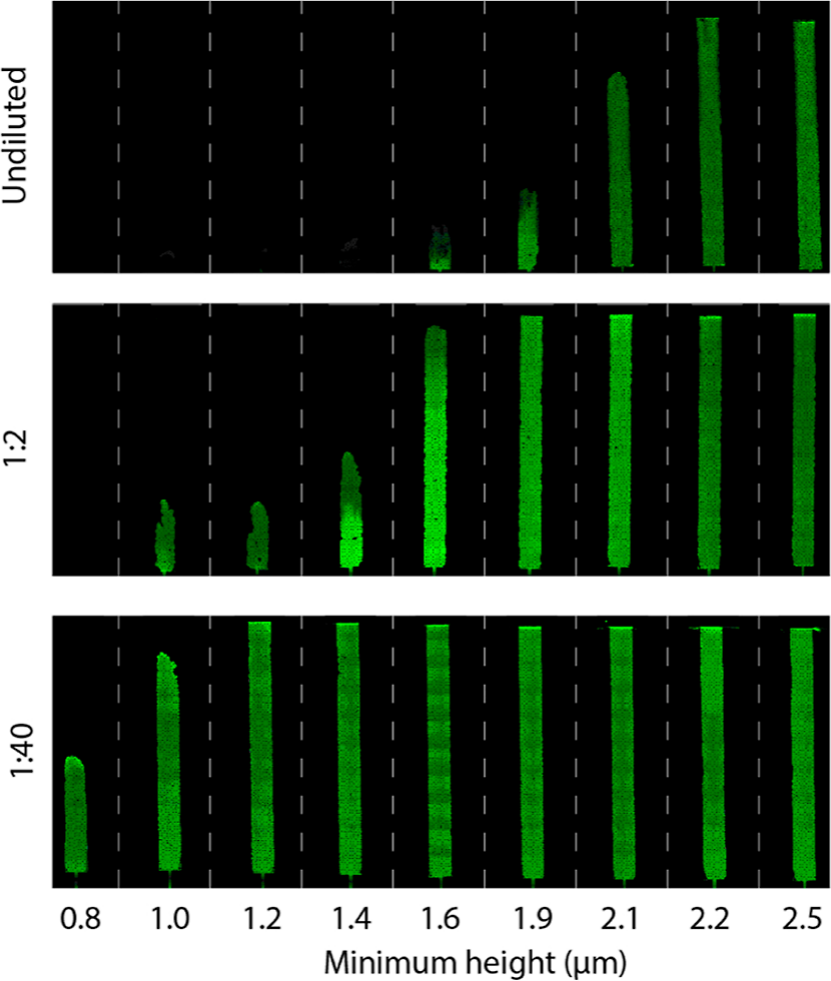
Fluorescence images of whole blood flowing through devices with different outflow heights. The channels shown have a width of 2 mm. The investigated dilutions were undiluted, 1:2, and 1:40 in PBS.

To enable preloading of biofunctionalized particles and to increase the device’s hydrophilicity,^37^ we treated the surfaces of both patterned and unpatterned PMMA sheets with poly(vinyl alcohol) (PVA) before sealing the device through UV/O-assisted thermal bonding (see Methods). Afterward, the devices were loaded with whole blood at different concentrations, and the filling factor in the CP region was monitored after 5 min (Figure 4). It was observed that undiluted blood does not reach the CP region in the devices with a shallower 3DR due to clogging effects. A notable change in the filling fraction of undiluted whole blood can be seen between channels with minimum depths of 1.6 and 1.9 μm. When an increased dilution is used, this transition in filling fraction is shifted toward smaller minimum channel heights.

**Figure 5:**
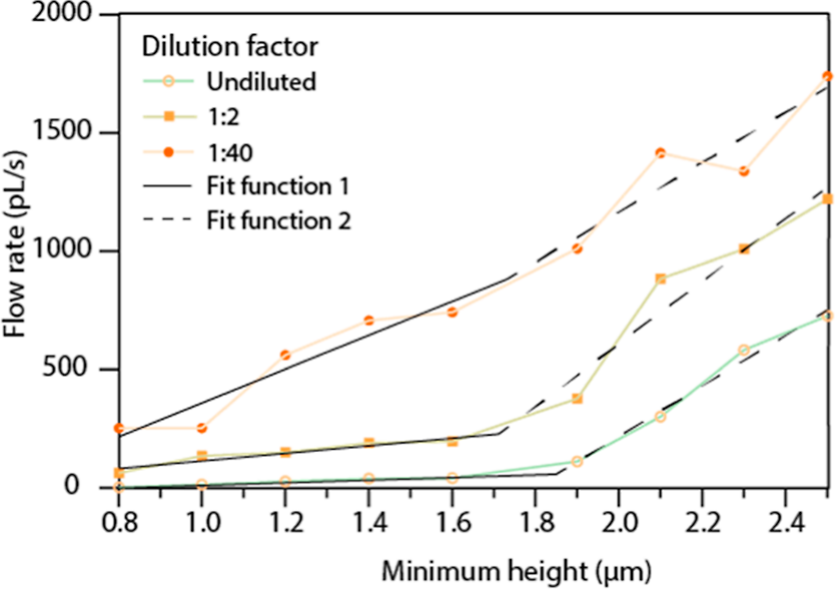
Filling velocity of the CP region at different concentrations of whole blood and various outflow heights. The data was fit with a segmental linear regression fit. The two fit functions are given by the solid and dashed black lines.

To evaluate this phenomenon more quantitatively, a time-lapse series was acquired at the beginning of the CP region to monitor the initial filling behavior of the devices with different dilution ratios (Figure 5). The obtained data were fitted with a segmental linear regression to estimate the critical height leading to a transition between flow regimes of clogging and unhindered flow. For more concentrated blood samples, the major transition in filling rate occurs between 1.6 and 1.9 μm. For a more dilute sample (1:40), the flow rate increases with an increasing minimum channel height. This is likely a result of an increased fluidic resistance with narrower channels, and RBCs apparently have a negligible role at such dilution rates.^48^ It is interesting to note that the aforementioned transition point is around the reported minimum thickness of RBCs of about 1.7 μm.^6,49^ These results may provide valuable insights into the blood transport microcapillaries, etc. Moreover, this transition region suggests that the RBCs are aligned at the constriction, which might be interesting for applications in combination with high-resolution microscopy. Based on these observations, we designed a new chip containing 9 devices with an equal minimal outflow height of 2.2 μm to ensure unhindered passage of RBCs.

### Rapid One-Drop Detection of Anti-BSA Antibodies Directly in Whole Blood

To address the shortcomings of both POCs and ELISAs, we used an optimized 3D microfluidic device to perform on-chip immunoassays directly in whole blood. This device allows for rapid readout and analysis of small sample volumes, while at the same time, it avoids numerous sample-handling steps. The immunoassay was constructed similarly to an indirect ELISA that targeted a specific antibody. We functionalized 2.8 μm streptavidin-coated particles with biotinylated BSA, acting as an antigen (Figure 1d). This was performed in PBS at a pH of 7.4. Then, the functionalized particles were diluted in distilled water and loaded into the device. After filling the CP region, the loaded devices were dried under vacuum for 1.5 h. Distilled water rather than PBS was used for the final particle dilution to prevent the formation of salt crystals and ensure proper and homogeneous drying of the device. The preloading of the functionalized particles is similar to the overnight plate functionalization in indirect ELISAs but requires almost 23 h less time while retaining a high degree of antibody stability.^35^

Once the device is duly prepared in advance, it is ready to be used for rapid and simple on-chip immunoassays. To showcase the capabilities of the device, we performed a proof-of-principle experiment in which a 2 μL PBS droplet containing diluted rabbit anti-BSA antibody as well as Cy5-conjugated donkey antirabbit antibody was applied onto the inflow region of the device. The former serves as a primary antibody against the antigen target, whereas the latter functions as a secondary fluorescent detection antibody.

**Figure 6:**
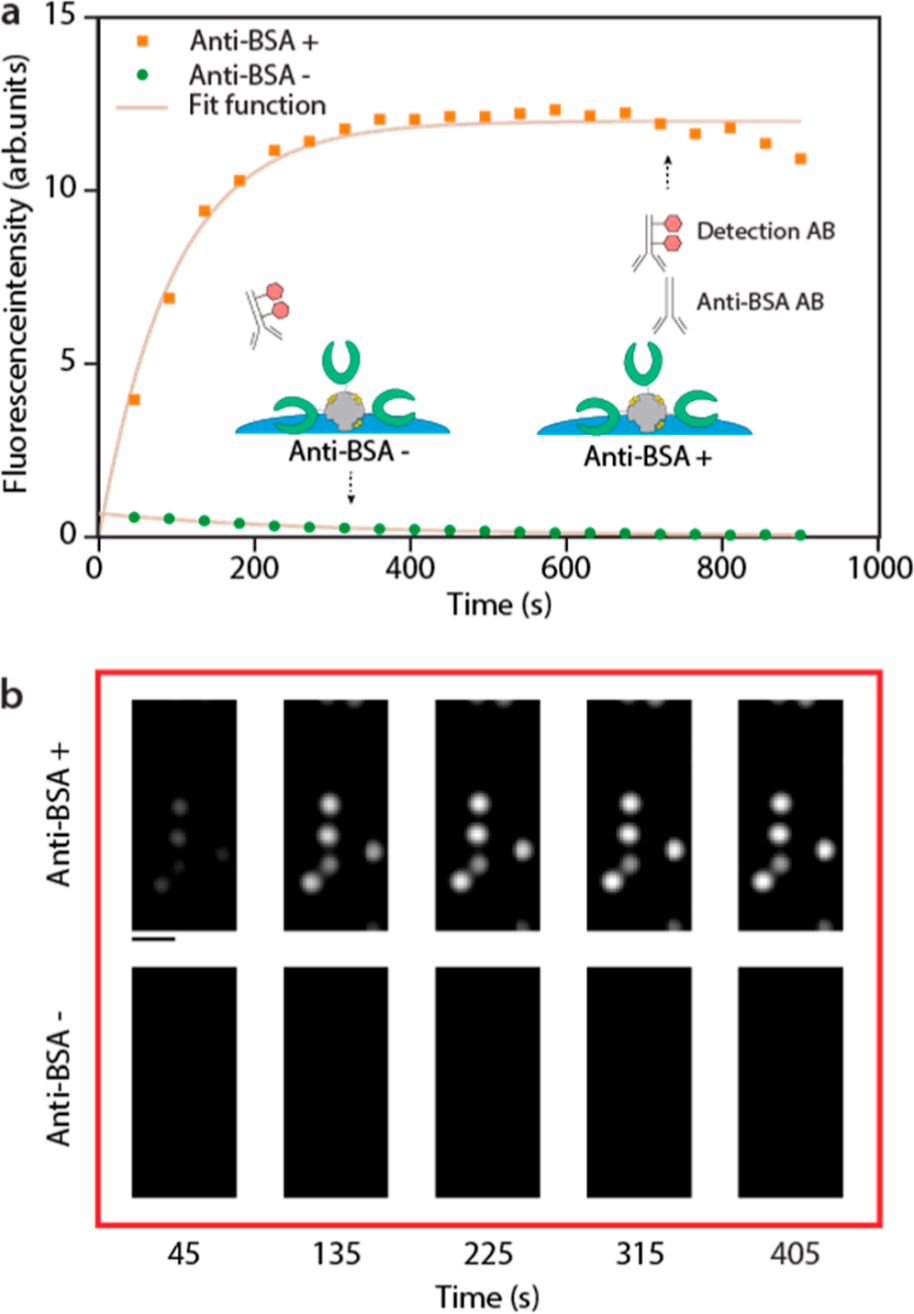
On-chip fluorescent immunosorbent assays in whole blood. (a) Time lapse showing changes in the fluorescent signal of the trapped particles over time, for both BSA+ and BSA– samples. (b) Fluorescent micrographs of BSA+ and BSA– samples at different times after device loading. The concentration of the secondary C5 donkey antirabbit antibody was 50 nM. The scale bar represents 10 μm.

The liquid was then passively drawn into the fluidic channel, flowing over the preimmobilized and BSA-functionalized particles. Due to the large surface-to-volume ratio due to the small size of the particles and reduced diffusion distances owing to the shallow dimensions of the channels, incubation time is substantially reduced in comparison to ELISA.^50,51^ This enables rapid binding of the primary anti-BSA antibodies to its antigen. Simultaneously, the secondary antirabbit antibodies conjugated with a fluorescent dye will bind to the primary antibodies on the particles’ surface. The secondary antibodies enable indirect fluorescence detection of the binding event between the primary antibody and its antigen. The accumulation of fluorescent signal on the particles was tracked with time-lapse series (Figure 6a), revealing a steady increase in signal, peaking around 360 s. A visual comparison of the fluorescent images (Figure 6b) shows a clear difference between positive and negative control samples already after 45 s. Additionally, in the negative control experiment, the fluorescent signal did not increase over time, evidencing the absence of nonspecific binding of the secondary antibody to the particle surface. On the contrary, a slight decrease over time was observed, likely due to a combination of bleaching of the autofluorescence of the particle as well as bleaching of the unbound secondary antibodies. The bleaching effect also became apparent after around 10 min in the case of the BSA-positive control sample. However, it should be mentioned that, based on the previously acquired flow rates, the CP is estimated to be filled after around 100 s, whereas the maximum signal was seen after 360 s. This suggests that not all of the available binding sites on the particles are saturated. Therefore, for future considerations, it would be beneficial for the devices’ sensitivity to enhance the capillary-pump’s capacity and thereby increase the volume of analyte that is flushed over the particles.

It is important to note that because of the operation principle of the device, no washing steps were performed, leaving unbound fluorescent detection antibodies in the channel. This results in an increased background signal that could potentially reduce the signal-to-noise ratio and, thereby, the LOD. Other microfluidic assays have addressed this issue by additional washing steps or complex predeposition antibodies to decrease the background signal over time and enable signal readout.^35^ However, this can be cumbersome and time-consuming. The presented device inherently mitigates this issue because of its shallow dimensions and small detection volume, which yields very low background signal, allowing to skip washing steps.^52^

### On-Chip Immunoassays Reveal a 2 nM LOD

**Figure 7:**
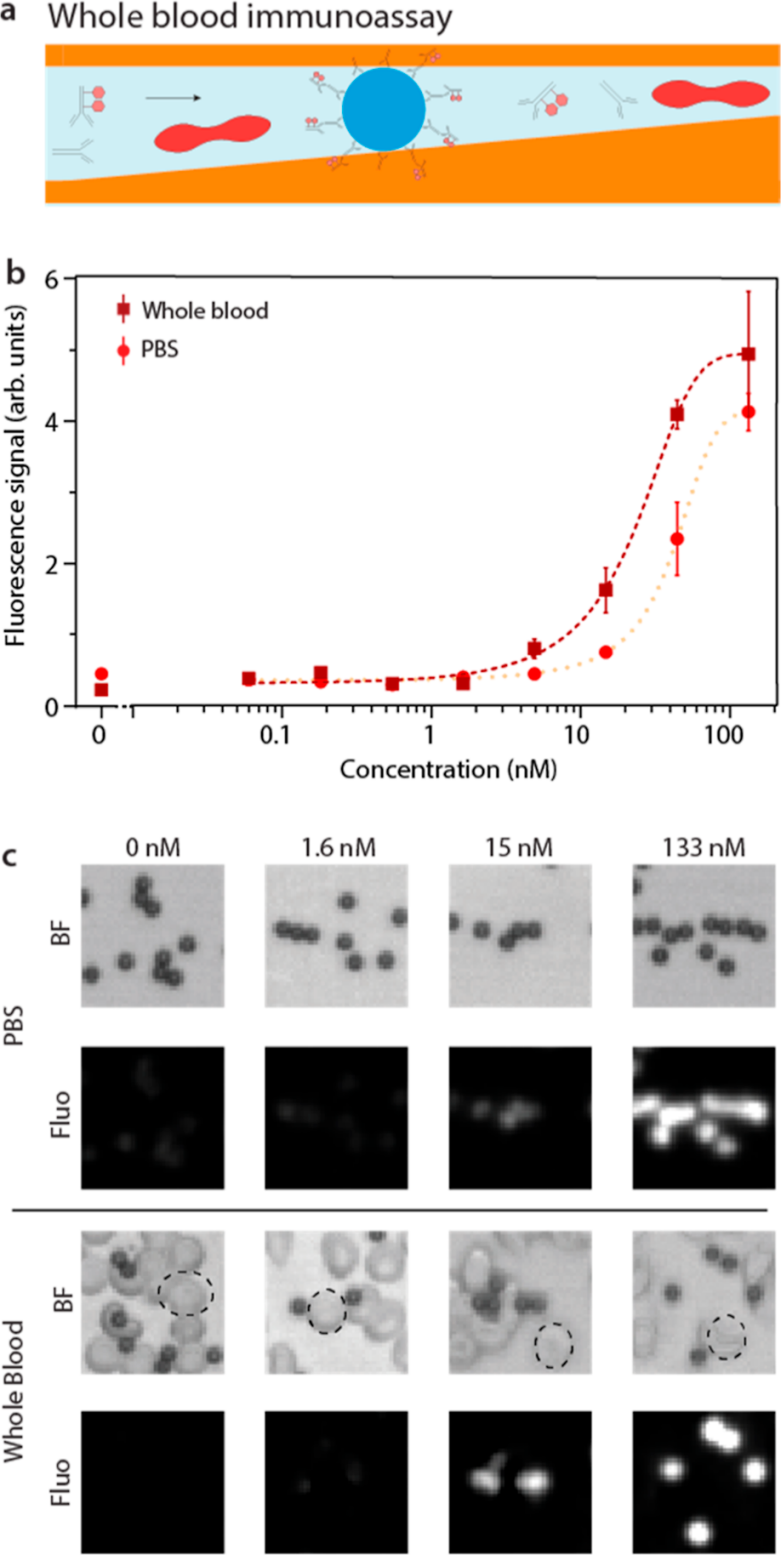
LOD of immunosorbent assays in whole blood and PBS. (a) Schematic of the immunoassay concept. (b) LOD curve showing the fluorescence intensity for different concentrations of anti-BSA antibody in diluted whole blood (1:40; red dotted line) and PBS (orange dotted line). (c) Bright-field and fluorescent micrographs of biofunctionalized particles in the TR when loaded with different concentrations of anti-BSA antibody in PBS (top) or whole blood (bottom). The RBCs are outlined by a dashed line. The scale bar represents 10 μm.

The previous section demonstrated the device’s capability to rapidly detect the presence of BSA-specific antibodies in a physiological buffer solution. As a next step, we benchmarked the whole blood assay against its LOD in PBS by determining the lowest detectable concentration of rabbit anti-BSA antibody while keeping the concentration of secondary detection antibodies constant. Each sample was allowed to flow over the preimmobilized particles for 6 min to ensure maximal binding of all the assay constituents (Figure 7a). Afterward, bright-field and fluorescent micrographs of the particles in the trapping region were acquired and the corresponding fluorescence intensity quantified (Figure 7c—top). The quantified data revealed an LOD of 11 nM in PBS (Figure 7b). Repeating the experiment with diluted whole blood (1:40) yielded a better LOD of 2 nM. This discrepancy can be possibly attributed to the blocking effect of serum constituents, which reduces nonspecific binding interactions^53^ This is evidenced by the higher signal of the PBS control in comparison to that in whole blood. Additionally, various other factors, such as pH^54^ or flow velocity,^55^ can play significant roles. The achieved LODs, both for PBS and whole blood, are in the relevant range for a variety of analytes^56^ and disease-specific antibodies.^57^

The bright-field micrographs of the whole blood samples (Figure 7c—bottom) show that RBCs are still present in the microfluidic channels. At this point, it is worth emphasizing that due to geometrical size constraints, the RBCs cannot cover the preimmobilized particles on their top and bottom surfaces. This means that from a top view, the RBCs cannot conceal the particles, which ensures that the fluorescent signal originating from the surface of the particles is not diminished by possible scattering and absorption effects.^58,59^ These experiments highlight that the LOD of BSA-specific antibodies is not negatively impacted by the presence of diluted whole blood.

### Passive Particle Size-Separation Enabling Facile Multiplexed Antibody Detection

**Figure 8:**
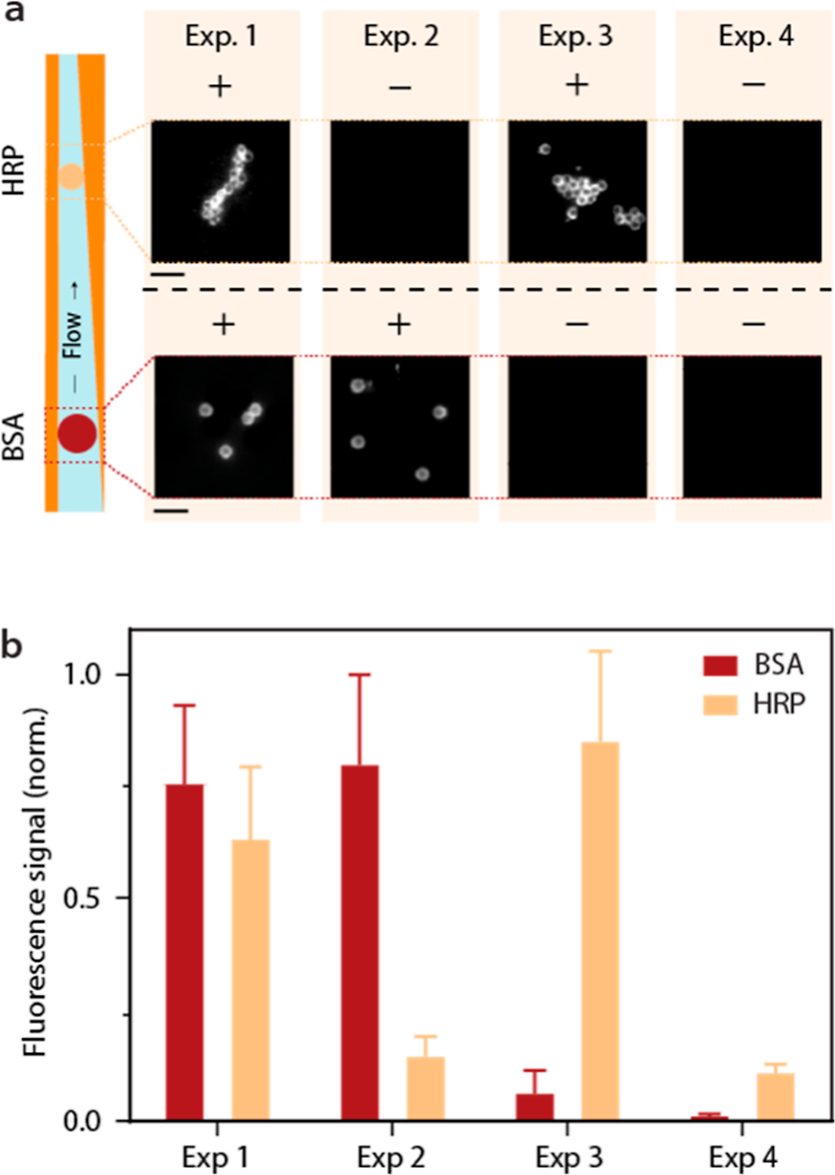
Multiplexed on-chip immunoassays. (a) Fluorescence images of 2 μm HRP and 2.8 μm biofunctionalized particles in different experimental conditions to show the multiplexing capabilities of the 3D microfluidic device. (b) Quantified fluorescent signal from particles shown in (a). The error bars represent the standard error of the mean (*N* = 3). All the scale bars represent 10 μm.

The passive size-dependent particle immobilization of the device has previously been shown to enable on-chip multiplexed detection of antibodies with distinct antigen targets.^37^ Here, we show that similar assays are also possible using whole blood assays. To do so, the shallowest point of the device was slightly reduced from 2.2 to 1.9 μm. This decrease in the minimum channel outflow height enabled the trapping of smaller 2 μm particles. The latter were functionalized with biotinylated HRP added to a suspension consisting of 2.8 μm BSA-functionalized particles. Subsequently, both particles were pretrapped in the trapping section of the 3DR, as explained previously. As a proof-of-principle experiment, diluted whole blood samples (1:40) containing controlled combinations of rabbit anti-HRP and rabbit anti-BSA antibodies were loaded into the device. The evaluation of the binding event of the primary antibodies with their respective antigen targets on the different particle sizes was enabled by the addition of a Cy5-conjugated fluorescent antirabbit detection antibody. After an incubation time of 6 min, fluorescent micrographs were taken at the positions inside the trapping section (Figure 8a). This is in contrast with other immunoassay technologies that leverage the same effect of decreased channel heights, where only one of the channel walls is biofunctionalized.^60^ The use of biofunctionalized particles in microfluidic channels increases the surface-to-volume ratio and decreases the diffusion distances, leading to a substantial reduction of incubation times.^61,62^ The quantification of the particle’s fluorescent signal is in agreement with the presence or absence of the respective primary antibodies (Figure 8b). More specifically, in exp 1 (BSA+/HRP+), a large fluorescent signal can be seen at both pretrapped biofunctionalized particles. However, when either the primary HRP (expt 2) or BSA (expt 3) antibody is removed, the obtained fluorescent signal at the relative trapping position drops sharply. If both primary antibodies are removed and only whole blood spiked with fluorescent detection antibodies is loaded into the device (exp. 4), the signal at the trapping positions drops even further. This evidences high specificity and low antibody cross-reactivity.

These results underscore that by using distinct biofunctionalized particles of different sizes, it is possible to perform on-chip multiplexed antibody detection against different antigen targets or diseases in whole blood samples. This is achieved while retaining a low sample volume and a rapid sample read-out. The reported device can deliver the results on a similar time scale as LFAs but provides the possibility for quantitative signal analysis, higher sensitivity, and multiplexing capabilities simultaneously.

## Conclusions

The results presented in this article show the feasibility of conducting immunoassays directly in whole blood by using capillary-driven microfluidic devices with a changing channel topography. The 3D profile inside the microfluidic channel proved to have a critical impact on the filling behavior of both undiluted and diluted whole blood samples. More specifically, it was found that a minimum channel height of 1.7 μm is required to enable the facile passage of the RBCs through the trapping region and to reach the CP. It was shown that an optimized 3DR enables efficient pretrapping of biofunctionalized particles, and due to the high surface-to-volume ratio, sample readout can be achieved in less than 10 min. Moreover, we highlight that the presence of whole blood does not negatively affect the LOD when compared to standard PBS. In addition, multiplexing capabilities in a series of proof-of-principle experiments while retaining very low volume requirements, rapid sample readout, and quantitative analysis have been demonstrated.

To increase the portability of the proposed on-chip immunoassay, our aim is to develop and use a smartphone-based fluorescence microscope. This technology has a proven track record in the field of fluorescent immunoassays, showing LODs in physiologically relevant ranges for a wide range of analytes and diseases.^63,64^ Additionally, we aim to further reduce the LOD, e.g., by enlarging the total volume of the CP in an effort to increase the sample volume and, with that, the number of bound antibodies on the biofunctionalized particles. Also, the use of brighter and more photostable fluorophores, such as quantum dots,^65^ or the addition of antifading chemicals^66^ could lead to a better signal-to-noise ratio.

On the device fabrication side, it will be interesting to investigate alternate combinations of nanofabrication methods to render the entire process even more cost-effective and up scalable. For instance, one could go from a 4 in. process to an 8 in. one and replace hot-embossing with high-throughput methods, such as roll-to-roll embossing^67^ or injection molding.^68^

The 3D microfluidic device further extends the use of the previously developed 3D nanofluidic device toward whole blood assays. For future research endeavors, it would be of interest to adapt it to various biofluids, such as saliva or urine, to increase the device’s versatility. Subsequently, by combining this with smartphone fluorescence microscopy, the technology has the potential to make a meaningful impact on the biomarker detection landscape.

## Abbreviations

CCR2: CC chemokine receptor 2
CCL2: CC chemokine ligand 2
CCR5: CC chemokine receptor 5
TLC: thin layer chromatography
